# Evaluation of Selected Properties of Dielectric Barrier Discharge Plasma Jet

**DOI:** 10.3390/ma16031167

**Published:** 2023-01-30

**Authors:** Michał Kwiatkowski, Piotr Terebun, Katarína Kučerová, Barbora Tarabová, Zuzana Kovalová, Aleksandra Lavrikova, Zdenko Machala, Karol Hensel, Joanna Pawłat

**Affiliations:** 1 Chair of Electrical Engineering and Electrotechnologies, Lublin University of Technology, 20-618 Lublin, Poland; 2 Faculty of Mathematics, Physics and Informatics, Comenius University, 842 48 Bratislava, Slovakia; 3 Institute of Plasma Physics of the Czech Academy of Sciences, Za Slovankou 3, 182 00 Prague, Czech Republic

**Keywords:** atmospheric pressure plasma, dielectric barrier discharge, plasma jet, plasma treatment of liquid, reactive oxygen and nitrogen species, biological materials, *Escherichia coli*

## Abstract

In the technological processes requiring mild treatment, such as soft materials processing or medical applications, an important role is played by non-equilibrium plasma reactors with dielectric barrier discharge (DBD), that when generated in noble gases allows for the effective treatment of biological material at a low temperature. The aim of this study is to determine the operating parameters of an atmospheric pressure, radio-frequency DBD plasma jet reactor for the precise treatment of biological materials. The tested parameters were the shape of the discharge (its length and volume), current and voltage signals, as well as the power consumed by the reactor for various composition and flow rates of the working gas. To determine the applicability in medicine, the temperature, pH, concentrations of H_2_O_2_, NO_2_^−^ and NO_3_^−^ and *Escherichia coli* log reduction in the plasma treated liquids were determined. The obtained results show that for certain operating parameters, a narrow shape of plasma stream can generate significant amounts of H_2_O_2_, allowing for the mild decontamination of bacteria at a relatively low power of the system, safe for the treatment of biological materials.

## 1. Introduction

Currently, one of the most important areas of the application of plasma technologies is the use of non-equilibrium plasma, where the energies of electrons are much higher than the energies of ions and neutral particles. The selectivity of energy and a low degree of ionization allow for chemical reactions initiated by high-energy electrons at a relatively low temperature of the working gas. In low-temperature plasma applications, systems with dielectric barrier discharge (DBD) are particularly popular, allowing to obtain a stable discharge without the presence of electric arcs. In the case of the treatment of biological and other heat-sensitive materials of various shapes, where it is necessary to limit the impact area and operate in ambient air, atmospheric pressure plasma jet (APPJ) systems are broadly used [1,2,3,4,5,6]. In these systems, the plasma generated inside the nozzle is blown towards the treated objects as a result of the forced gas flow. Particular systems of APPJ may differ in terms of the type of power supply, the discharge geometry and the composition of working gas. Due to the presence of a dielectric barrier, the reactors are powered by AC (including radio and microwave frequencies) or impulse voltage ranging from several to a dozen kilovolts. To obtain the lowest possible gas temperature while sustaining stable discharge, mixtures with the majority of noble gas (helium, argon) are used, and the power of the system rarely exceeds a dozen watts. Indirect treatment is often used in order to lower the temperature even further, in which the treated biological material is not in direct contact with the plasma, but rather with active particles carried along with the gas stream (afterglow effect).

In medicine, DBD APPJ reactors have been used for the deactivation of pathogens [5,7,8,9,10,11,12,13,14], wound healing [9,14,15,16], stomatology [14,17,18] and anti-tumor treatment [14,19,20,21]. Examples of other low-temperature applications include surface modification [3,22,23,24] or improving agricultural seed germination [25,26,27]. Several effects of plasma treatment, such as UV-radiation and electric fields, are employed in the treatment of biological materials, but the most important of these remains the effect of reactive oxygen and nitrogen species (RONS) [28,29,30]. Plasma operating in atmospheric air in contact with liquids can generate significant amounts of various RONS in both gas and liquids phases, which are relevant for biological and environmental applications.

Advanced oxidation processes employ several kinds of environmentally friendly oxidants in the technological process allowing for superposition effect. Thus, they are considered the most efficient way of the removal of impurities, bactericidal decontamination and activation of surface. Electrical discharge can be generated directly in the treated liquid or in the gas intrusions in the liquid phase. Moreover, plasma can interact with the liquid indirectly when the discharge occurs in gaseous phase in the vicinity of the liquid surface and then active plasma species are further transferred via the surface to the bulk of the liquid [31,32,33,34,35,36,37,38,39,40,41,42,43,44,45,46,47,48,49,50,51,52,53].

When the discharge occurs in a multiphase environment, the nature of activated species strongly depends on actual composition of the liquid and the gas. Chemical processes that take place in the electrical discharges in water include direct formation of reactive radicals such as hydroxyl (•OH), hydrogen (H), superoxide (O_2_•^−^), perhydroxyl (HO_2_•) and oxide anions, and molecular species such as hydrogen peroxide (H_2_O_2_) and ozone (O_3_). Nitrogen-based species are also produced, such as nitrogen oxide radicals (NO•, NO_2_•), nitrate (NO_3_^−^) and nitrite (NO_2_^−^) anions, peroxynitrite (ONOO^−^), and also nitric (HNO_3_), nitrous (HNO_2_) and peroxynitrous acids (ONOOH) (Table 1).

The group of Brisset et al. [39,52,57,58,59] investigated humid air plasmas and electrical discharges generated in contact with the surface of liquids. In such an environment, RONS derive from N_2_, O_2_ and H_2_O, therefore hydrogen peroxide H_2_O_2_, ozone O_3_, or nitrogen oxides NO_x_ are expected to form, although O_3_ is not favored by the presence of water. Emission spectroscopy measurements of plasma in humid air revealed that •OH and NO• radicals are simultaneously present in the discharge, with a much higher density for strongly oxidizing •OH radicals than for NO• radicals. The later ones are known as parent molecules for acid derivatives HNO_2_ and HNO_3_, inducing a rapid pH lowering of the solution. Thus, the presence of both •OH and NO• radicals can enhance the efficacy of the treatment process [57,60,61,62,63,64,65].

Plasma formed species are mainly considered with plasma–liquid interactions and induce acidification and oxidation reactions. The acidification effect is related to the formation of transient nitrous acid HNO_2_ (which disproportionates into NO and nitric acid for pH < 6) and stable nitric acid HNO_3_. A weak peroxynitrous acid ONOOH (pKa = 6.8) is also formed under certain conditions. The oxidizing character of plasma treatments is mainly attributed to •OH, H_2_O_2_ and ONOOH. Apart from these basic reactions, peroxynitrous acid ONOOH and its matching ion, peroxynitrite ONOO^−^, react as nitrosating and nitrating agents on double bonds and carboxylic acids. That makes them the key agents for bacterial inactivation because of their chemical attack at the microorganism membranes [39,59,66].

The aim of this study is to determine the optimal operating parameters and the possibility of generating active plasma species for plasma medical applications in helium DBD APPJ powered by radio frequency power supply. The tested parameters are discharge shape (its length and volume), electrical signals for different composition and flow rates of the working gas. To investigate the possibility of using the reactor in medicine, the temperature and concentrations of selected RONS (H_2_O_2_, NO_2_^−^, NO_3_^−^) in non-buffered and buffered liquids are examined, as well as the effect of the plasma treatment on the reduction of *E. coli* bacteria in these liquids.

## 2. Materials and Methods

**Figure 1:**
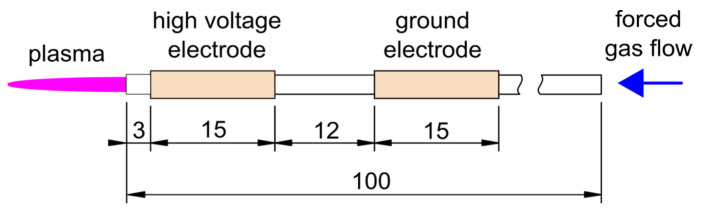
Geometry of DBD APPJ reactor, dimensions are given in mm.

The tested reactor was the DBD APPJ reactor in a system with two ring-shaped electrodes wrapped around a ceramic tube of 1.5/2.5 mm inner/outer diameter. Plasma was generated inside a tube and then directed towards the treated object by the forced gas flow, as shown in Figure 1.

**Figure 2:**
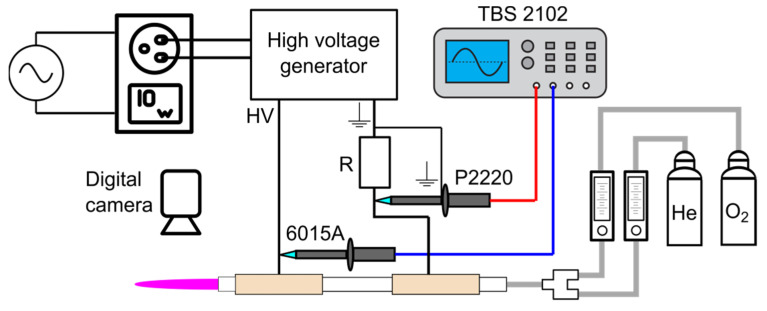
Experimental setup for measuring electrical quantities.

The reactor was powered by a 20 kHz high-voltage power supply using a fly-back transformer. The voltage between electrodes *U_2_* was measured with a high-voltage probe (Tektronix P6015A, Berkshire, UK) connected to a digital oscilloscope (Tektronix TBS 2102, Berkshire, UK). The total current *I_2_* was measured indirectly by measuring the voltage (Tektronix P2220, Berkshire, UK) across a 100 Ohm low-inductive resistor (ARCOL AP101 R100, Munich, Germany), as shown in Figure 2. In order to compare the efficiency of the system for various conditions, the power *P_1_* and *S_1_* at the input of the power supply was also measured with a wattmeter (Wattman, HPM-100A, AD POWER Co., Ltd., Bucheon-si, Republic of Korea).

Helium and its mixture with oxygen were used for all experiments. During the tests, gases were fed through glass tube flow meters (Brooks Instrument SHO-RATE, Hatfield, PA, USA). The widest range of flow rates (0.32–5.8 L/min) was used in the study of the influence of the working gas on the shape of the discharge, which was carried out on the basis of the length of the glowing part of the plasma depicted on photos (Nikon D7000, Bratislava, Slovakia; 1/80 s exposure time) and known dimensions of reference points.

A study on the efficiency of generating selected compounds in aqueous phase and the decontamination effect on *Escherichia coli* was performed for two liquids: simulated tap “water” (NaH_2_PO_4_ solution, pH ~ 5, σ ~ 600 μS/cm) and 2 mM “PB” (phosphate buffer solution, pH ~ 7, σ ~ 560 μS/cm). The concentrations of selected species (H_2_O_2_, NO_2_^−^, NO_3_^−^) were determined by the colorimetric method described in our previous publication [67], using reagents and absorbance wavelength presented in Table 2. For the verification of the results, the standard error of the mean from two repetitions were used.

**Figure 3:**
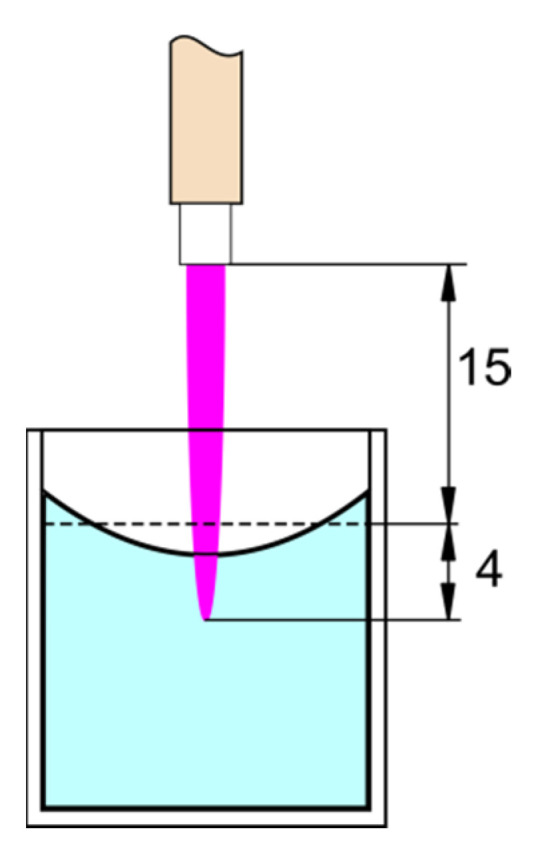
Experimental setup for chemical and biological treatment. The plasma jet impinges on the liquid surface and slightly dives into the liquid.

During the measurements, 2 mL of the liquid was placed in a 24-well plate at 15 mm distance between the nozzle and the treated liquid sample (Figure 3). Measurements were performed for 2 and 5 min of plasma treatment. The reference (control) samples were tested for 5 min action of the gas directed to the surface of the liquid. The temperature of the liquid was measured immediately after plasma treatment using an uninsulated K-type thermocouple with an electronic temperature compensation meter (Yu Ching Technology DT-847U), noting the highest meter reading within 10 s.

For the same distance, biological treatment was performed for planktonic *E. coli* (CCM3945) dissolved from gel disc in “water” or “PB”. A pellet of *E. coli* was suspended in 10 mL of desired aqueous solution and let at 35 °C for 18 h, then 5 ml of this suspension was added to 45 ml of “water” or “PB” solution. The obtained initial concentration of bacteria was ~10^6^–10^7^ CFU/mL (colony forming unit per milliliter). After plasma treatment, the sample was diluted and cultivated on agar plates overnight. The control samples were processed the same way except the (discharge) plasma was not applied. Then colonies were counted and logarithmic reduction was evaluated as a difference of CFU for controls and samples. For statistical interpretations, median and interquartile range from 4 repetitions were used.

In addition, measurements of pH were performed by pH-meter.

## 3. Results

### 3.1. Discharge Images

**Figure 4:**
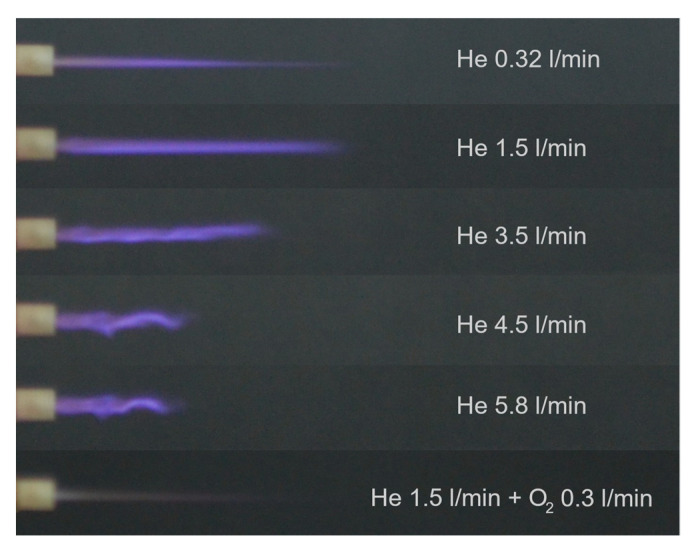
The shape of the discharge for different gas flow rates and composition of the mixture.

Examples of the discharge images for different gas flow rates are shown in Figure 4. A comparison of the obtained results shows a very strong influence of the flow rate value on the discharge shape, which becomes turbulent for too high flow rates (>3.5 L/min). The highest discharge volume was obtained at a flow rate of 1.5 L/min and this value was chosen as the reference for the following studies. The addition of oxygen admixture to He resulted in a reduction in the length and volume of the discharge, therefore an oxygen addition of 0.3 L/min was selected for further measurements, for which a similar discharge length was still obtained.

### 3.2. Electrical Characteristics

**Figure 5:**
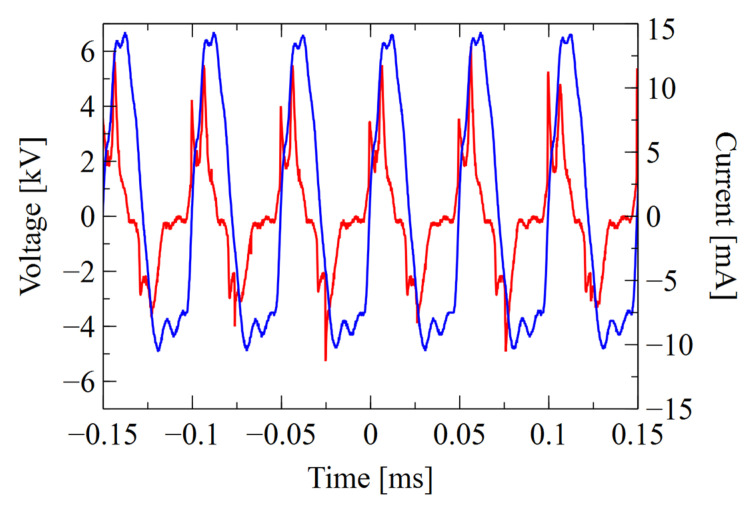
Waveform of voltage (blue line) and current (red line) for 1.5 L/min helium.

**Figure 6:**
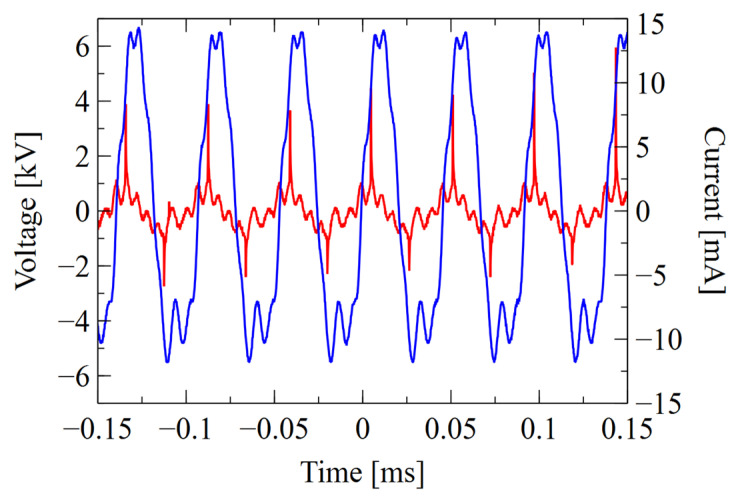
Waveform of voltage (blue line) and current (red line) for 1.5 L/min helium and 0.3 L/min oxygen mixture.

The waveforms of the voltage *U*_2_ signal and the current *I_2_* drawn from the high-voltage power supply are shown in Figure 5 and Figure 6.

The measurement results and the calculated active (P) and apparent (S) power values are summarized in Table 3. Active power (P) refers to the part of power that is absorbed by the load (plasma) and usually is smaller than the apparent power (S) that is a product of voltage and current. For both gas compositions, the system is capacitive. In the case of mixture of helium and oxygen (He+O_2_), despite similar voltages, the total current in the system is several times lower, which is also reflected in the power consumed by the supply system. For this system, based on the power ratio, a significant decrease in the efficiency of the power supply can also be noticed.

### 3.3. Chemical and Bilogical Analysis

Prior to plasma treatment, the liquids were kept at room temperature (20 °C). For all tested conditions, the liquid temperature after plasma treatment did not exceed 25 °C.

**Figure 7:**
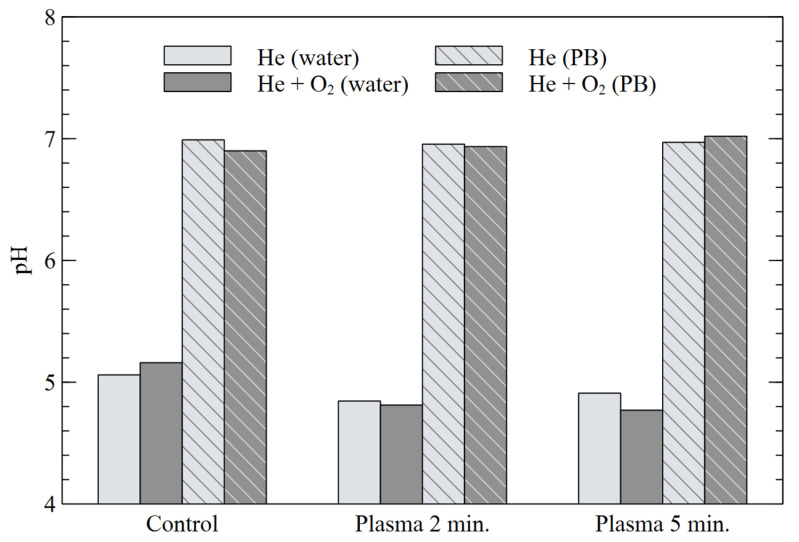
Measurements of pH of water and PB for different plasma treatment times.

Figure 7 shows pH values of the liquids after the 2 and 5 min treatments. Plasma treatment resulted in a slight decrease of pH value of “water”, which is more noticeable with a mixture of He+O_2_. For the “PB” solution, no significant difference in pH was observed even after 5 min of treatment. These values are consistent with our formerly-obtained results [7,38,39,52]. Gaseous nitrogen oxides NOx were generated in plasma jet surrounded by ambient air and further transported to the liquid phase, where nitrate NO_3_^−^ and nitrite NO_2_^−^ anions accompanied with hydronium ions H_3_O^+^ were formed. Consequently, reaction of NO_2_^−^ with H_3_O^+^ ions resulted in an acidic product: nitrous acid HNO_2_, which caused a slight pH decrease of “water”. The aim of the phosphate buffer (PB) solution was to investigate the pH impact on the other parameters of plasma-treated liquids. Presented results confirm that phosphate buffer was not affected by plasma.

**Figure 8:**
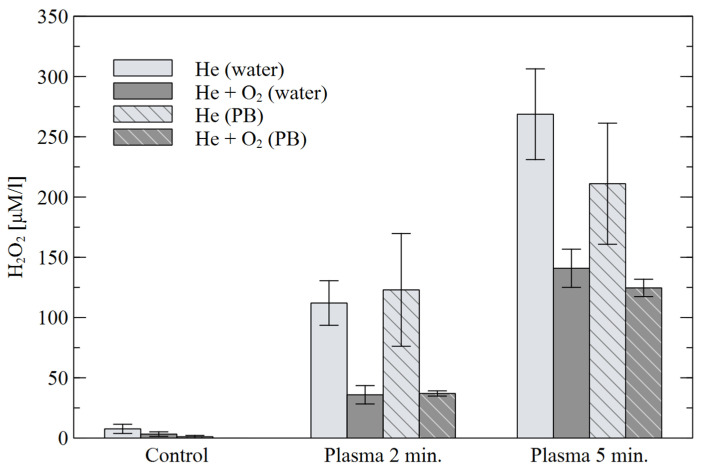
Concentration of H_2_O_2_ in water and PB for different plasma treatment times.

**Figure 9:**
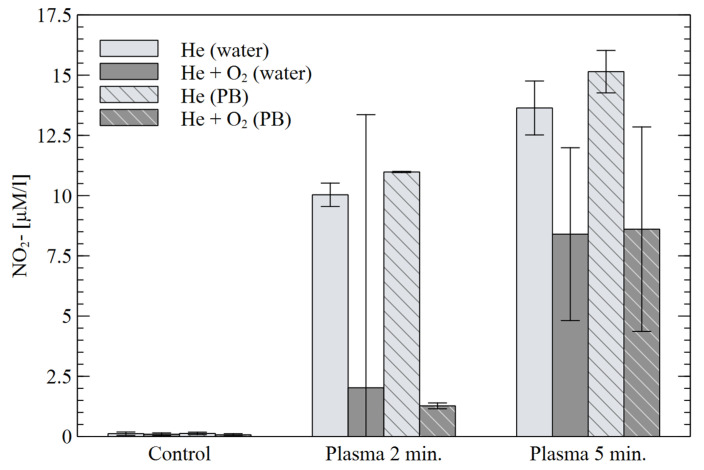
Concentration of NO_2_^−^ in water and PB for different plasma treatment times.

The results of the H_2_O_2_ concentration in the solutions are shown in Figure 8. For both treatment times, a much higher concentration was observed for He alone than for its He+O_2_ mixture, although the results for the second mixture were more reproducible. The presence of the buffer did not significantly affect the results, except for the “water” 5-min treatment with He alone, for which the highest concentration was obtained. The relationship between H_2_O_2_ concentration and treatment time is almost linear, however, for NO_x_^−^ compounds, the chemistry of the chemical reactions is not so simple. Figure 9 shows the results obtained for the NO_2_^−^ concentration. Much significant and more reproducible changes were observed in He alone. In this case, slightly higher concentrations are also seen for the “PB” solution. Increasing the treatment time from 2 to 5 min resulted in a slight increase in the concentrations in He, and over triple increase in the NO_2_- concentration for the mixture of He+O_2_.

**Figure 10:**
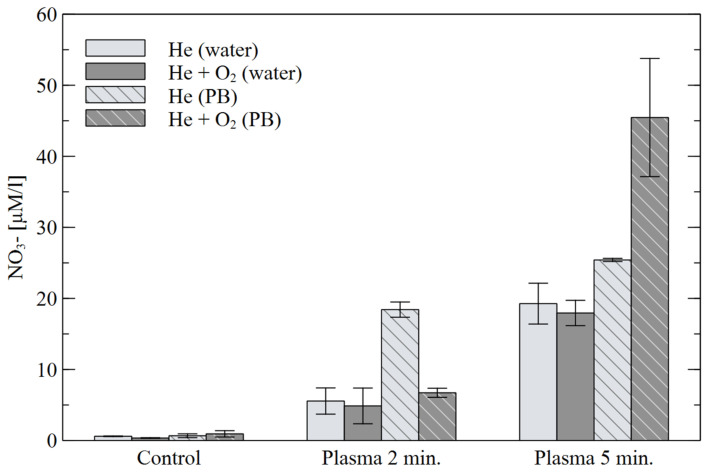
Concentration of NO_3_^−^ in water and PB for different treatment times.

The concentration of nitrates NO_3_^−^ for the 2- and 5-min plasma treatments is shown in Figure 10. For both working gases, the concentration of NO_3_- is higher for the “PB” liquid, while for the mixture of He+O_2_ the difference is more than double. In this case, the addition of oxygen caused a much greater amount of NO_3_^−^ compared to He alone.

**Figure 11:**
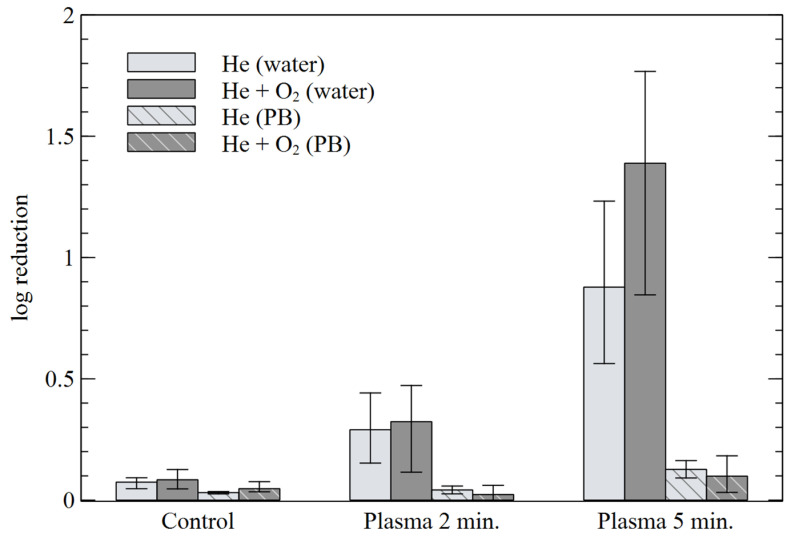
Logarithmic reduction of *E. coli* in aqueous water and PB solutions for different treatment times.

Figure 11 shows the effect of plasma treatment on the reduction of *E. coli* suspended in water or PB. Better antibacterial results were obtained for the mixture of He+O_2_. However, the observed bacterial reduction for both mixtures is significant but relatively small. For both gases, the use of the “PB” solution significantly lowered the treatment efficiency.

## 4. Discussion and Conclusions

The DBD APPJ discharge images show a strong influence of the working gas on the shape (length, volume) of the generated plasma. The most important factor influencing the shape of the plasma jet is the gas flow rate, which is also often observed in the literature [2,68,69,70,71]. A small addition of oxygen, introduced to increase the amount of RONS, decreased the length of the glowing part of the discharge. It may be related to a lower degree of ionization for non-noble gas admixture, however the reduction of the amount and mobility of charges and a visible decrease of the discharge current did not significantly change the shape of the discharge itself, which had a narrow shape suitable for focalized point treatment of biological objects.

When analyzing the measured voltage and current signals, apart from the phase shift related to the capacitive nature of the load, a certain asymmetry of the half-periods of signals can be observed. The current peaks associated with micro-discharges, due to the large differences between individual gas mixtures, have a higher magnitude and less phase shift for the positive part of signal than for the negative part, which is well visible in He working gas. The time intervals between the current and voltage peaks also differ, which indicates the influence of the accumulated charge on the dielectric surface. Uneven charging may be related to the geometry of the system and plasma propagation in the direction of the gas flow. It can cause both a different value and number of micro-discharges in particular cycles [72,73,74]. In the case of the used power supply, the discharge current peaks also influence the shape of the voltage between the electrodes, which dropped significantly in time intervals corresponding to micro-discharges.

Much lower values of the peak current obtained for the oxygen-containing mixture were also reflected in the power and efficiency of the system. For a mixture of He+O_2_, the active and apparent power drawn from the source is much lower than that for He alone, but utilization of the active power part used by the load is much lower. It is related to the lower value of the micro-discharge current with the same value of the displacement current, which reduces the overall efficiency of the entire system.

Due to the low discharge currents, the power of the system for both tested gas mixtures was relatively low. This is reflected in the temperature of the treated liquid, which only slightly increased after the 5 min treatment. Despite the low power, the reactor allows for the generation of H_2_O_2_, NO_2_^−^ and NO_3_^−^ and their transport to the liquid phase. In the case of hydrogen peroxide H_2_O_2_, the relationship between the concentration and the treatment time is almost linear. In terms of the amount of generated H_2_O_2_, this DBD APPJ system is more energy-efficient than the mini-gliding arc reactor tested in the past with the same diagnostic methods [67]. This may be related to the better transport of the active species along with the plasma stream which is in contact with the liquid and the possibility of generating H_2_O_2_ directly in it.

On the other hand, the amount of generated NO_x_ compounds is relatively small, which is also consistent with only a slight pH drop. Despite the possibility of interaction with nitrogen N_2_ present in the atmospheric air surrounding the discharge [75], plasma occurs mainly in the working gas, the components of which are transferred along with the stream to the liquid but the energy delivery is much weaker here than in more powerful plasma discharges, e.g., mini-gliding arc or transient spark [7,67]. Due to the small concentrations of nitrogen compounds and the more complex chemistry involved in both the liquid reactions and the reagents used to determine their concentrations, the results are not as well reproducible as for H_2_O_2_. The obtained low concentrations, however, may have contributed to the high H_2_O_2_ content, through preventing the decomposition of H_2_O_2_ by its reaction with NO_2_- [76].

The obtained RONS concentrations also allowed the reduction of *E. coli*, which may be related to the decontamination effect of H_2_O_2_ [30,77]. However, the pH-dependence of the effect even for a slight pH drop to 4.8 in water most likely indicates an additional role of ONOOH formation in bacterial reduction, via the reaction of H_2_O_2_ and low concentration of NO_2_^−^. The log reduction of bacteria here is lower than other DBD APPJ reactors [78,79] but similar to the results obtained in the liquid phase [77]. Due to the low power of the reactor and the narrow shape of plasma stream, these results seem to be sufficient for the practical application of tested DBD-APPJ in medicine where microorganisms need to be selectively inactivated without harming the healthy tissue cells. Examples include precise biomedical treatments, such as wound healing, oral cavity infections or focalized tumor treatments. An additional advantage of the presented low-power DBD APPJ is also the indirect nature of the treatment, where the discharge taking place mainly between the electrodes of the reactor and the target is mildly treated by its reactive effluent, thus preventing the complexity and danger of applying high voltage directly to the biological targets, where a similar plasma jet reactors are the subject of worldwide research [30].

## Figures and Tables

**Table 1:** Properties of selected oxygen base species [39,54,55,56].

Species	Formula	Standard Electrochemical Potential [V]	pH Where Present	Role
Hydroxyl radical	•OH	+2.59	pH < 11.9	strong oxidant
Hydrogen peroxide	H_2_O_2_	+1.77	pH < 11.6	strong oxidant, weak reductant
Superoxide anion	O_2_•^−^	−0.33	pH > 4.8	weak reductant
Perhydroxyl radical	HO_2_•	+1.49	pH < 4.8	strong oxidant
Hydroperoxide anion	HO_2_^−^	0.88	pH > 11.6	weak oxidant, weak reductant
Singlet oxygen	^1^O_2_			
Ozone gas	O_3_	+2.07		strong oxidant
Atmospheric oxygen	O_2_	+1.23		weak oxidant
Solvated electrons	e_(aq)_^−^	−2.77	pH > 7.85	strong reductant
Nitrate anion	NO_3_^−^			oxidant in acidic solutions
Nitrite anion	NO_2_^−^			oxidant in reductant
Peroxynitrite	ONOO^−^			strong oxidant
Nitric oxide radical	NO•			reductant oxidant
Nitrogen dioxide radical	NO_2_•			Oxidizing agent reducing agent

**Table 2:** Conditions for colorimetric method.

Compound	Reagents	Maximum Absorption
H_2_O_2_	TiOSO_4_ solution	407 nm
NO_2_^−^	Griess assay	540 nm
NO_3_^−^	Enzymatic reduction to NO_2_^−^ + Griess assay	540 nm

**Table 3:** Electrical parameters of the reactor for used gas mixtures.

Mixture	*P*_1_ [W]	*U*_2 *RMS*_ [kV]	*I*_2 *RMS*_ [mA]	*P*_2_ [W]	*S*_2_ [VA]
He	11.09	4.15	4.25	10.24	17.67
He+O_2_	4.6	4.17	1.38	2.96	5.76

## Data Availability

Data available upon justified request after contact with authors.
